# Individual exposure to *Simulium bites and intensity of Onchocerca volvulus infection*

**DOI:** 10.1186/1756-3305-3-53

**Published:** 2010-06-18

**Authors:** C A Jacobi, P Enyong, A Renz

**Affiliations:** 1 Tropenmedizinisches Institut der Universität Tübingen, Wilhelmstraße 27, D-72074 Tübingen, Germany; 2 Medical Research Station Kumba, PO Box 55, Republic of Cameroon; 3 Current Address: Institut für Evolution und Ökologie, Abteilung Vergleichende Zoologie, Universität Tübingen, Auf der Morgenstelle 28, D- 74074 Tübingen, Germany; 4 Universitätsklinikum Magdeburg, Klinik für Gastroenterologie, Hepatologie und Infektiologie, Leipzigerstr. 44, 39120 Magdeburg, Germany

## Abstract

**Background:**

*Onchocerca volvulus*, the causative agent of river blindness, is transmitted through the black fly *Simulium damnosum *s.l., which breeds in turbulent river waters. To date, the number of flies attacking humans has only been determined by standard fly collectors near the river or the village. In our study, we counted the actual number of attacking and successfully feeding *S. damnosum *s.l. flies landing on individual villagers during their routine day-time activities in two villages of the Sudan-savannah and rainforest of Cameroon. We compared these numbers to the number of flies caught by a standard vector-collector, one positioned near the particular villager during his/her daily activity and the other sitting at the nearest *Simulium *breeding site.

**Results:**

Using these data obtained by the two vector-collectors, we were able to calculate the Actual Index of Exposure (AIE). While the AIE in the savannah was on average 6,3%, it was 34% in the rainforest. The Effective Annual Transmission Potential (EATP) for individual villagers was about 20 fold higher in the rainforest compared to the savannah.

**Conclusions:**

Here we show for the first time that it is possible to determine the EATP. Further studies with more subjects are needed in the future. These data are important for the development of future treatment strategies.

## Background

In the human population of hyperendemic onchocerciasis villages, a high individual parasite load results from an intense and prolonged exposure to the transmission of infective larvae of *Onchocerca volvulus *by the black fly *Simulium *vectors. Assessing heterogeneity in exposure may contribute to the ongoing debate about the processes generating overdispersion of parasite loads among hosts, namely, the fact that a majority of hosts harbour no or light infections while a few are heavily infected. Its quantification is important for the development of mathematical descriptions of parasite transmission dynamics. In West Africa, persons particularly at risk are fishermen, farmers and children who spend much of their daytime near *Simulium damnosum *s.l. infested rivers. The intensity of transmission is commonly assessed using standardized procedures with a standard fly collector, catching all simuliid female flies which come to feed on his fully exposed legs [1-4]. This number is referred to as the daily biting rate (DBR). Dissecting these flies will give us the number of infective L3 larvae = daily transmission potential (DTP). This method, although useful in providing data on *Simulium *landing rates and *O. volvulus *transmission potentials, cannot be used to calculate the actual number of infective larvae transmitted to individual villagers. Heterogeneity in exposure among villagers is likely to result from age- and sex-dependent variation in behaviour and occupation, the use of protective clothing, and individual variability in their attractiveness to flies [4]. There is also a marked differential dispersal of the flies inland away from the river in the two main bioclimatic zones: parous (and therefore potentially infective) flies tend to remain close to the river in the savannah whilst in the rainforest they disperse far from the river in search of a blood meal [5].

The objective of this work was to determine the Actual Index of Exposure (AIE) to *Simulium *flies and effective transmission of *O. volvulus *in the two bioclimatic zones. Since we did not dissect the flies we caught, we were not able to determine the DTP from our data, thus we used the data from [6] and from [7]. Here we present the results from a small survey conducted in the rainforest and the savannah of Cameroon. In addition to just counting the number of flies coming to land on the "test-person" in the savannah, we also determined the proportion of flies that started probing blood and the proportion of flies killed before or at the end of feeding, i.e., before or after infective larvae might have escaped from the flies. The determination of the AIE is possible and large scale studies are needed in the future to generate these important data.

## Results

In the rainforest, the *Simulium *landing rate as determined by the fly collector situated near the observed person was between 70 to 141% of the landing rate near the river at the *Simulium *breeding site (Table 1; row: f/g ). At the river Dilolo, the Daily Biting Rate (DBR) was on average 195 flies/person-day, near the person the DBR was 174. On average 116 flies (67% of the DBR near the person) were recorded to land on the "test-person" (Table 1; row: a, f, g). During their normal day time activity, these "test-persons" were exposed to a *Simulium *biting rate between 0 and 190% of the landing rate of the nearby sitting fly collector (Table 1; row: a/f). Using the data from [7] we are able to calculate the Effective Transmission Potential (ETP, i.e. the effective number of larvae transmitted to a person per unit time). In the rainforest, the average Daily Biting Rate at the river in 1999 was 435 flies/person-day and the average number of *O. volvulus *larvae was 0,064 L3/fly. The observed average index of exposure was 34% (Table 1; row: j) and the ETP amounts therefore to 9,45. L3 larvae/person-day (435 × 0,064 × 0,34) resulting in an average EATP of 3450 L3 larvae/person-year.

**Table 1:** Individual exposure of villagers to S. damnosum s.l. in the rain forest and savannah.

		SAVANNAH			RAINFOREST			
**Age/Gender**	**Row**	**50/m**	**54/f**	**35/m**	**60/m**	**11/m**	**19/f**	**63/m**
								
		**S.K.**	**N.A.**	**O.S.**	**C.K.**	**J.D.**	**P.D.**	**S.T.**

Occupation		nightwatch	farmer	farmer	farmer	school boy	farming	farmer
Clothing^1^		lss, lt	sk, lss	lss, lt	lss, lt, sh, so	sh	sk, sss	lt, sss
Protection by clothing^1 ^(estimate)		60%	30%	60%	100%	40%	30%	60%
**Microfilarial load at iliac crest (mf/mg)**		**10**	**16**	**25**	**1**	**49**	**149**	**44**
**Number *Simulium *landing per day**	**a**	8	4	25	0	269	82	114
proportion of flies that -								
- flew away unfed	b	.33	.33	.55	.0	.64	.15	.16
- sucked successfully	c	.44	.07	.11	.0	.31	.12	.53
- were killed before sucking	d	.0	.0	.10	-	n.d.	n.d.	n.d.
- were killed after feeding	e	.23	.60	.24	-	n.d.	n.d.	n.d.
**Flies per day on standard fly collector**								
- caught near the "test-person"	f	10	9	21	222	141	157	175
- caught at the river banc	g	172	40	89	158	202	183	237
Standard daily biting rate (at river)^2^	h	**328**			**435**			
Standard daily transmission potential^2^	I	**6,6**			**27,8**			
								
**Actual Index of Exposure^3 ^(AIE)**	**j**	**3%**	**6%**	**10%**	**0%**	**89%**	**22%**	**25%**
**Effective Annual Transmission Potential^4^**	**365ij**	**72**	**144**	**239**	**0**	**9031**	**2232**	**2537**

Results from three days of observation per "test-person".

^1^: Clothing (estimate degree of protection; from [4]): lss - long sleeved shirt (5%); sss - short sleeved shirt (0%); lt - long trousers (30%); sh - shoes (30%); so - socks (30%); sk - skirt (20%);

^2^: Standard ABR and ATP, measured by the fly-collector at the river banc; data from [6] and [7].

^3^: Proportion of the biting rate on the person as compared to the biting rate on the fly collector at the river: j = a (c+e)/g in the savannah and a (c+k)/g in the rainforest; k: average of e (from savannah) = .36.

^4^: O. *volvulus *infective larvae per person and year i

In the savannah, the *Simulium *landing rate near the "test-person" was much lower than in the rainforest, between 6 to 23% of the landing rate near the river as was determined by the fly collector (Table 1; row: f/g). According to our data, on average the DBR at the Vina du Sud was 100 flies/person-day, near the "test-person" the number was 13 as determined by the standard fly collector. On average twelve flies (92% in comparison to the fly collector) were recorded to fly to the observed person. In summary, only 8 of 37 feeding flies completed their blood meal successfully and could thus maintain the transmission cycle of onchocerciasis (Table 1; row: c). The portion of flies killed was divided into flies killed before or at the end of their blood meal. Using published data from [6] we were able to calculate the ETP. In the savannah, the Daily Biting Rate in Galim was on average 328 flies/person-day. Our observed average index of exposure of the villagers was 6,3% (Table 1; row: j). Since two L3 larvae have been found in every 100 flies, it gives an average EATP of (328 × 0,02 × 0,063 × 365) 151 L3 larvae/person-year in the savannah.

## Discussion

Quantitative data on the intensity of effective transmission and its relationship to the ensuing intensity of infection are a prerequisite for effective monitoring of onchocerciasis control programmes [8,9]. Existing data are based on an assessment of DBR and DTP that probably overestimates the values as they are based on the standard vector collector. These persons are fully exposed from dawn to dusk, usually sitting at a place near the river, where the *Simulium *flies are most abundant. A more realistic estimate of the effective DBR and DTP can be obtained by catching flies at different localities around and in the villages, where the villagers spend most of the day-time, and by measuring the Daily Visiting Frequency and exposure of different age and sex groups [4,10]. However, this still does not allow us to quantify the individual exposure of single villagers and to correlate it with their parasite load as measured by the microfilarial density in the skin. In view of the wide variation of individual parasite load observed in endemic villages, more precise data on effective ATP would be useful. These can be used to develop mathematical models of onchocerciasis [11-13] and for assessing the effect of density-dependent factors involved in the regulation of the host-parasite interaction. A significant deviation from proportionality between inoculation rate (effective ATP) and worm load (microfilarial load) would indicate the operation of density-dependence, and could be due to factors operating upon parasite establishment and/or parasite survival among others.

The maximum exposure by the standard fly collector is used as an index of the landing/biting rates of *Simulium *flies in a particular locality. Here we show, that this method is inadequate to reflect true levels of exposure as indicated by a variable degree of individual clothing by the villagers (which was 40 and 70% in the savannah and between 0 to 70% in the rainforest) and by a variable degree of feeding success by the flies. For instance, the 60 year old male farmer C.K. (Additional File 1) always had worked and works fully protected against *Simulium *bites, hence he is very little exposed and his microfilarial load at the iliac crest is only 1 mf/mg (Table 1). In this study, about one third of the flies which came to the "test-persons" flew away, without probing blood. Another third sucked blood and managed to fly away. The last third was killed by the person, before or after sucking blood. Thus, only a third of the flies which come to a person may actually contribute to the transmission of onchocerciasis, even though the fraction killed after the blood-meal may also contribute to the transmission to that single person. However, this proportion also depends on the *S. damnosum *subspecies: For example, in Liberia, only 25% of females of *S. yahense *fed successfully while in Togo this proportion was 75, 45 and 44% of *S. soubrense*/*sanctipauli*, *S. damnosu*/*sirbanum *and *S. squamosum*, respectively [14]. In the present study in Cameroon the flies belong to the rainforest and savannah strains of *S. squamosum*. The reason why our daily biting rates are lower than the ones published [6,7] are unknown. Seasonal variation, environmental factors and the low number of collection days can be discussed. The EATP in the rainforest is more than 20 fold higher than the EATP in the savannah. However, the microfilarial density in the skin is only 5 times higher in the rainforest than it is in the savannah. Several reasons can be discussed. Indeed, there might be density regulated reaction towards the parasite in the body. As a certain number of microfilaria is reached, the immune system will respond quite strongly in eliminating the microfilaria [15]. In the different bioclimatic zones, different strains of the vector as well as of the parasite might be present. Thirdly, especially in the savannah, the *Simulium *vectors are also carrying larvae from *Onchocerca *species other than *O. volvulus*, like *Onchocerca ochengi *or *O. dukei*. These will stimulate the immune system without contributing to the worm load [16,17]. Of course, our sample size of seven subjects is very small, but the aim of this study was to first find out whether or not this data can be collected. Further studies with more subjects are in urgent need.

Obviously, the presence of an observer and the need to routinely check for feeding flies might have changed the behaviour of the persons so that the proportion of flies killed during the blood meal might not represent true values. However, from the observation of other persons (mainly children), who were standing near the observer (at the beginning of these experiments) and who were not directly involved, a similar frequency of swapping feeding flies could be observed. Thus only about one third of the flies will keep up the transmission cycle. Much higher success rates may occur on animal blood hosts, especially on cattle, which may be twice as attractive for the flies: on this host most the flies fed on the inguinal region, where only very few were disturbed or even killed [17].

## Conclusions

The aim of this study was to determine the Actual Index of Exposure (AIE) and the Effective Annual Transmission Potential (EATP) of individual villagers in two different bioclimatic zones in Cameroon. This data is compared to the maximal exposed vector collector at the river and near the "test-person". The EATP in the rainforest is about a 20 fold higher than in the savannah. Additionally we determined that only about 30% of the flies which come to the individuals may contribute to the transmission of onchocerciasis. Thus it should be important to be aware of these data when developing mathematical models for transmission of onchocerciasis. However, larger studies with more subjects are needed.

## Methods

**Figure 1:**
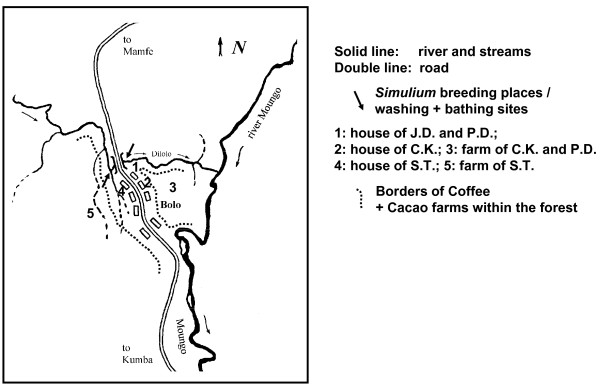
**Surroundings of Bolo**.

**Figure 2:**
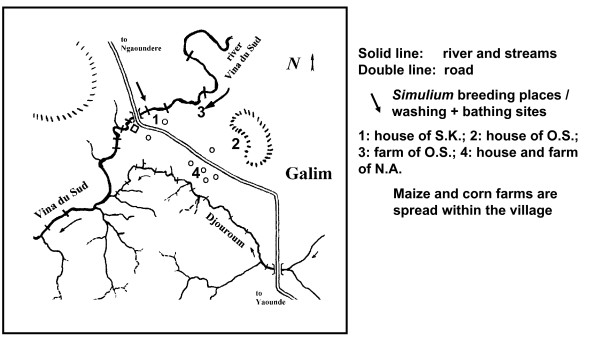
**Surroundings of Galim**.

Observations were carried out in localities situated in the rainforest (Bolo, Additional Files 2 and 3) and the savannah (Galim, Additional File 4) regions of Cameroon, Africa. In the rainforest, the survey was performed in the village of Bolo (4° 52'N × 9° 28'E), located near the Dilolo river (Additional Files 5 and 6), a tributary of the river Mungo (Figure. 1). There, onchocerciasis is hyperendemic with a microfilarial (mf) prevalence of 95% and an arithmetic mean mf density of 60 mf/mg of skin (all ages), reflecting a very intense transmission (Annual Biting Rate (ABR) = 158775 flies/person-year, Annual Transmission Potential (ATP) = 10162 L3 *O. volvulus *larvae/person-year, data from [7]). In the savannah, the study was conducted near the village of Galim (7° 13'N × 13° 34'E), situated at the river Vina du Sud (Figure 2, Additional File 7). The ABR at the river bank was 119720 and the ATP was 2,394 [6].

Four persons in the rainforest and three persons in the savannah (Additional File 8: "test-person" OS) were observed during their day-time activities in the rainy season, when transmission is high (Table 1). These individual villagers ("test-persons") of different sex, age and occupation were selected upon their willingness to collaborate, and were asked to give their consent. The observation was carried out at a distance (about 2 meters) from the "test-person" from where the blackflies could still be seen on the skin with the naked eye, but care was taken not to obstruct the person. Each observation lasted between three to four consecutive days and was carried out from dawn to dusk (7 am to 6 pm). The degree of coverage of their body by the person's clothing, the duration of their stay in different places and the number of flies coming to bite (landing rate) and successfully feeding (biting rate) were recorded. This was done by asking the person to check their body every 1 to 3 minutes for any feeding *Simulium *fly. In addition, all exposed body parts were observed by C.A.J. to make sure that nearly all (presumably more than 90%) incoming flies were recorded by him. Additionally, the location of the fly landing was recorded by C.A.J. and note was taken as to whether or not the fly flew away, sucked blood or was killed.

As controls, standard fly collectors were positioned near the river bank, where transmission is the highest, and also near the "test-person". These maximally exposed persons collect *Simulium *flies from dawn to dusk and are the positive controls.

## List of abbreviations used

ABR: Annual Biting Rate; AIE: Actual Index of Exposure; ATP: Annual Transmission Potential; DBR: Daily Biting Rate; DTP: Daily Transmission Potential; EATP: Effective Annual Transmission Potential; ETP: Effective Transmission Potential; Mf: Microfilariae.

## Competing interests

The authors declare that they have no competing interests.

## Authors' contributions

CAJ selected the "test-persons", instructed the fly collectors and collected the data in the field. CAJ and AR wrote this manuscript. PE was of great help in the field and helped gather some recent data on the *Simulium *biting rate in Bolo. The ideas for this work was developed by AR, he was supervising each phase of this project.

## Supplementary Material

Additional file 1 **"Test-person" CK in his working gear**. Pictures from Bolo and Galim: the village, villagers and the breeding sites are shown. Click here for file

Additional file 2 **The village of Bolo, main road**. Pictures from Bolo and Galim: the village, villagers and the breeding sites are shown. Click here for file

Additional file 3 **Children of Bolo**. Pictures from Bolo and Galim: the village, villagers and the breeding sites are shown. Click here for file

Additional file 4 **The village of Galim, main road**. Pictures from Bolo and Galim: the village, villagers and the breeding sites are shown. Click here for file

Additional file 5 **Breeding site at the river Dilolo**. Pictures from Bolo and Galim: the village, villagers and the breeding sites are shown. Click here for file

Additional file 6 **Children working at the river Dilolo**. Pictures from Bolo and Galim: the village, villagers and the breeding sites are shown. Click here for file

Additional file 7 **Breeding site at the Vina du Sud**. Pictures from Bolo and Galim: the village, villagers and the breeding sites are shown. Click here for file

Additional file 8 **"Test-person" OS with his family in front of their home**. Pictures from Bolo and Galim: the village, villagers and the breeding sites are shown. Click here for file
