# Supplementary figures and images for: Individual exposure to Simulium bites and intensity of Onchocerca volvulus infection
Source: Parasit Vectors. 2010 Jun 18;3:53. doi: 10.1186/1756-3305-3-53 (PMC2910011; doi:10.1186/1756-3305-3-53)

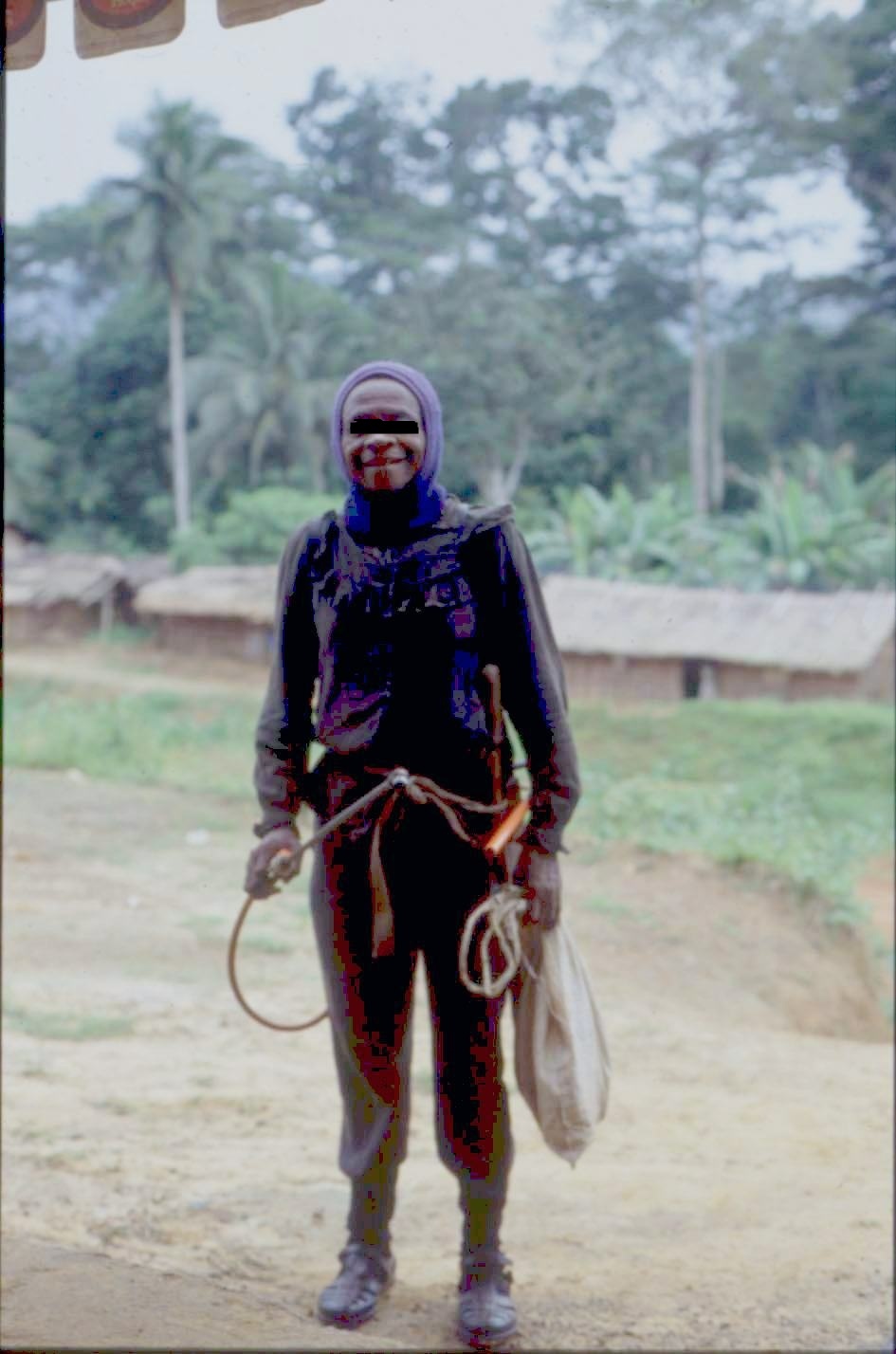

Supplement: Additional file 1 — "Test-person" CK in his working gear. Pictures from Bolo and Galim: the village, villagers and the breeding sites are shown. [file 1756-3305-3-53-S1.JPEG]

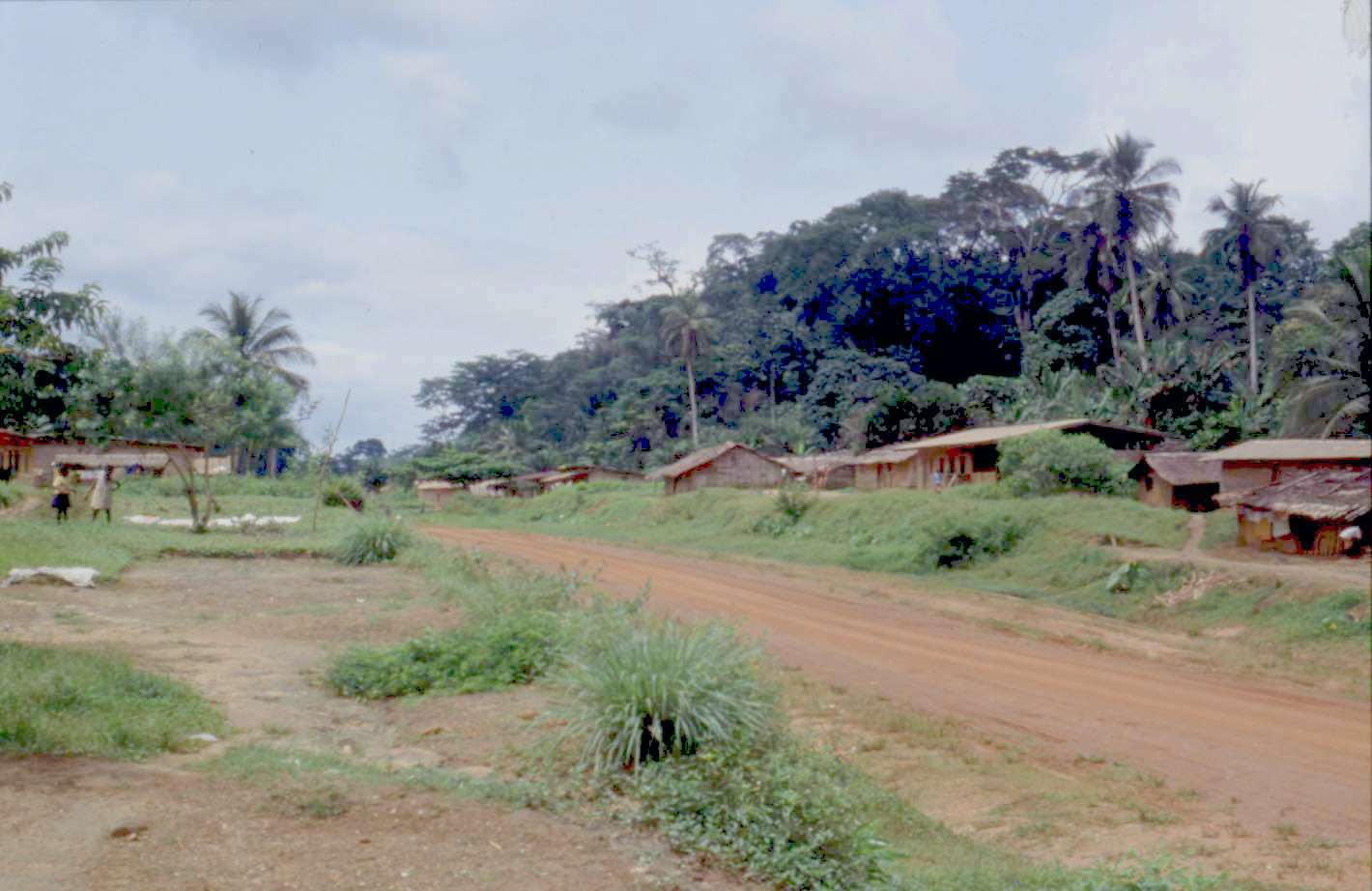

Supplement: Additional file 2 — The village of Bolo, main road. Pictures from Bolo and Galim: the village, villagers and the breeding sites are shown. [file 1756-3305-3-53-S2.JPEG]

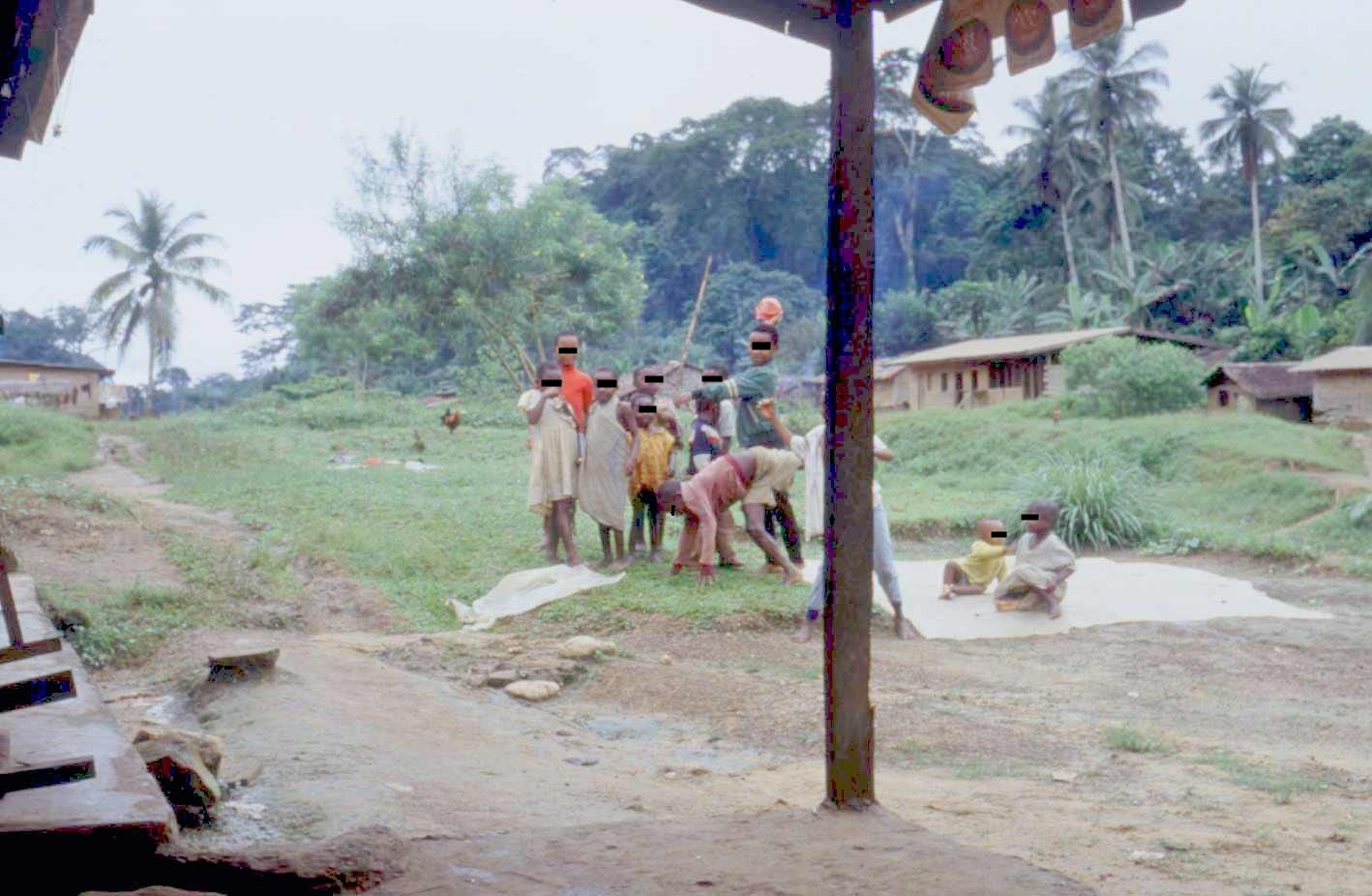

Supplement: Additional file 3 — Children of Bolo. Pictures from Bolo and Galim: the village, villagers and the breeding sites are shown. [file 1756-3305-3-53-S3.JPEG]

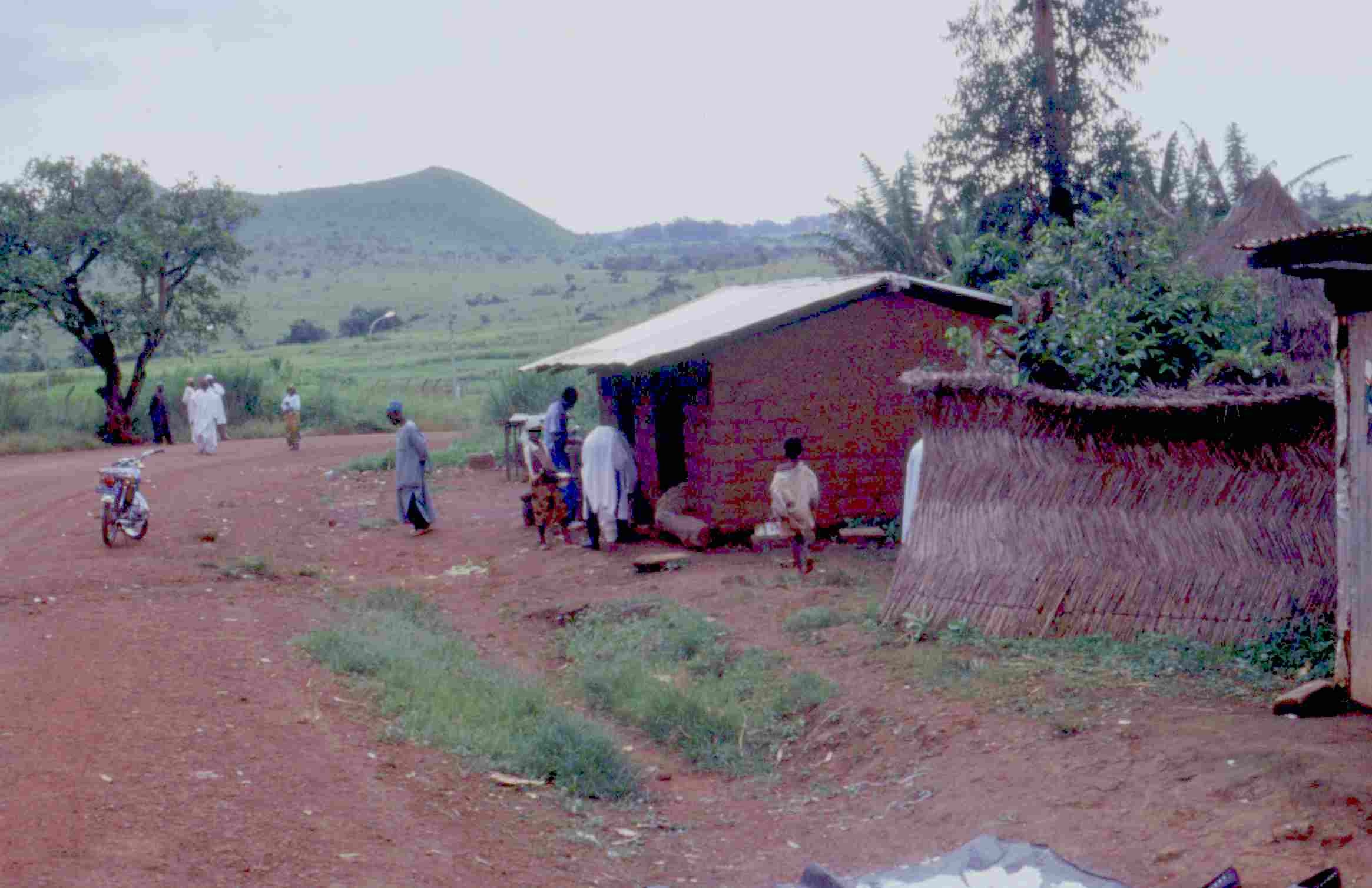

Supplement: Additional file 4 — The village of Galim, main road. Pictures from Bolo and Galim: the village, villagers and the breeding sites are shown. [file 1756-3305-3-53-S4.JPEG]

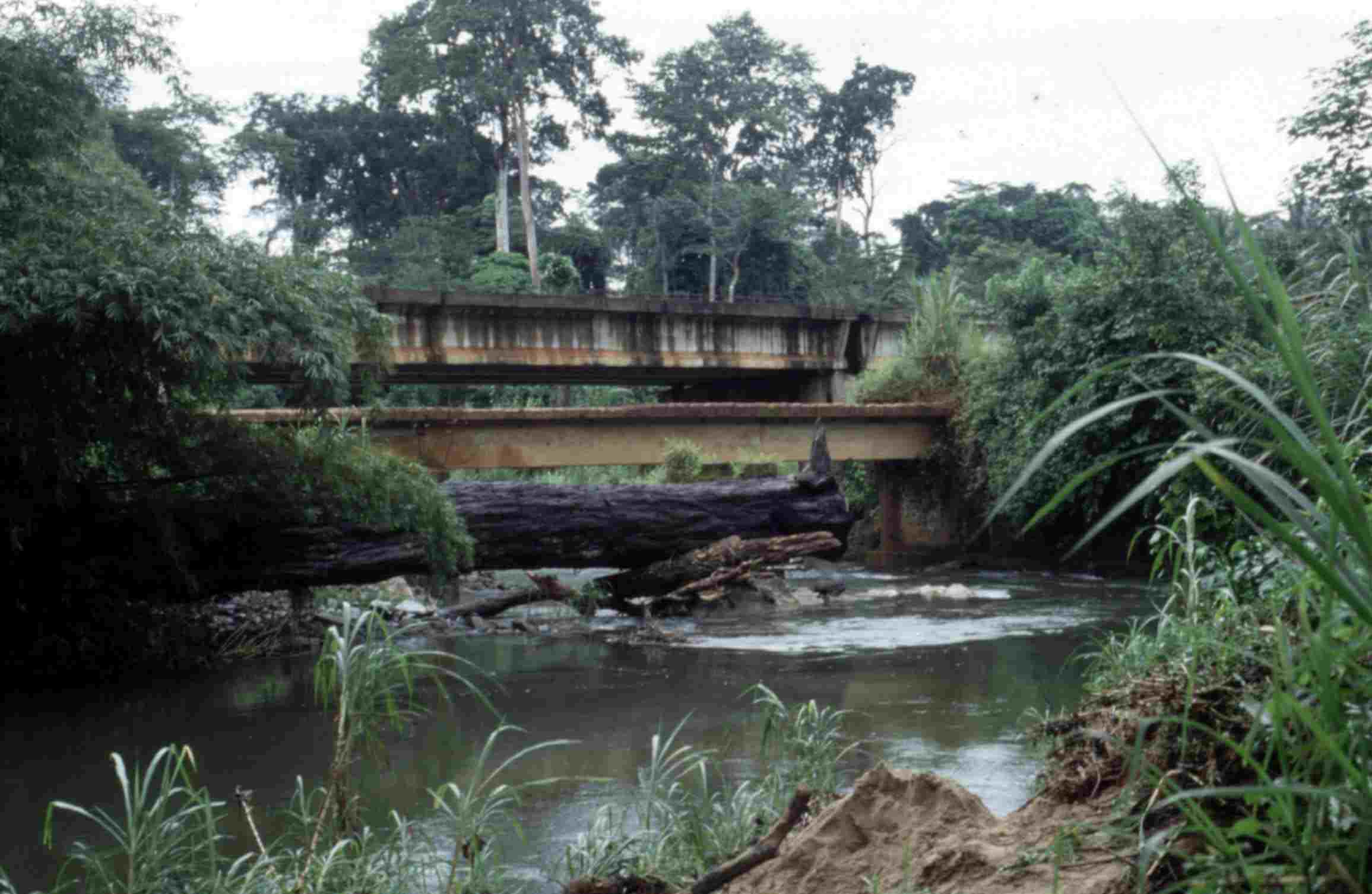

Supplement: Additional file 5 — Breeding site at the river Dilolo. Pictures from Bolo and Galim: the village, villagers and the breeding sites are shown. [file 1756-3305-3-53-S5.JPEG]

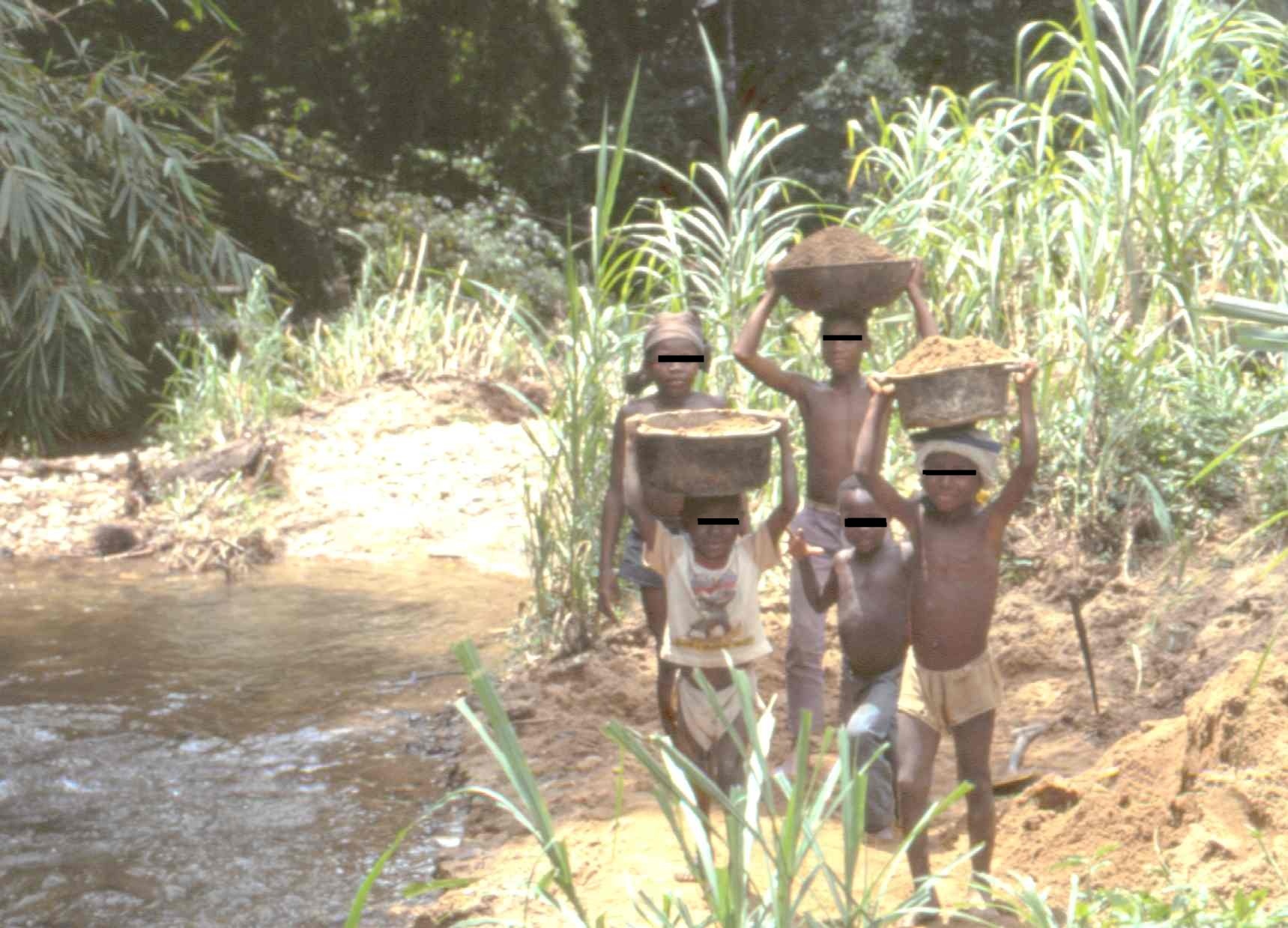

Supplement: Additional file 6 — Children working at the river Dilolo. Pictures from Bolo and Galim: the village, villagers and the breeding sites are shown. [file 1756-3305-3-53-S6.JPEG]

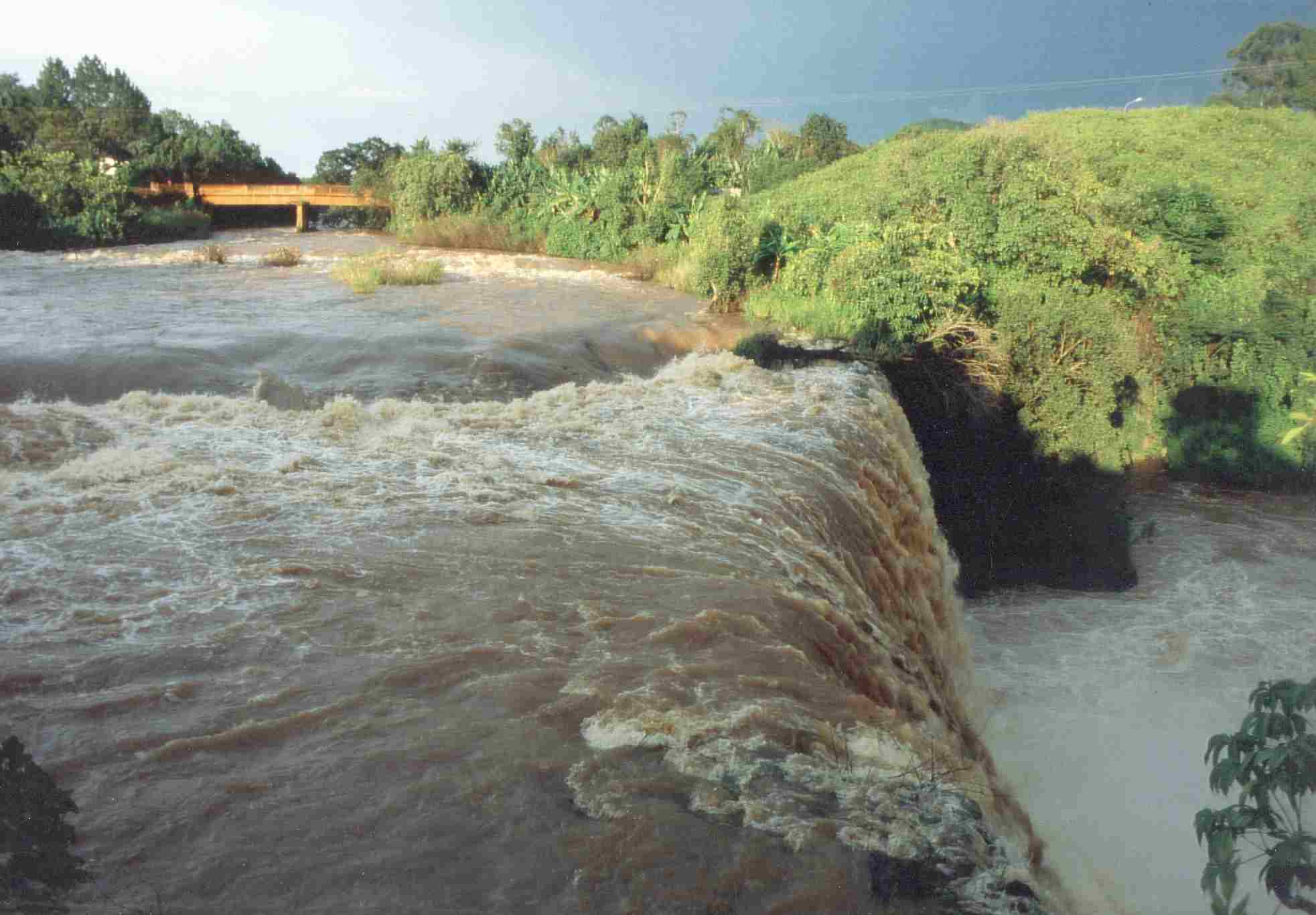

Supplement: Additional file 7 — Breeding site at the Vina du Sud. Pictures from Bolo and Galim: the village, villagers and the breeding sites are shown. [file 1756-3305-3-53-S7.JPEG]

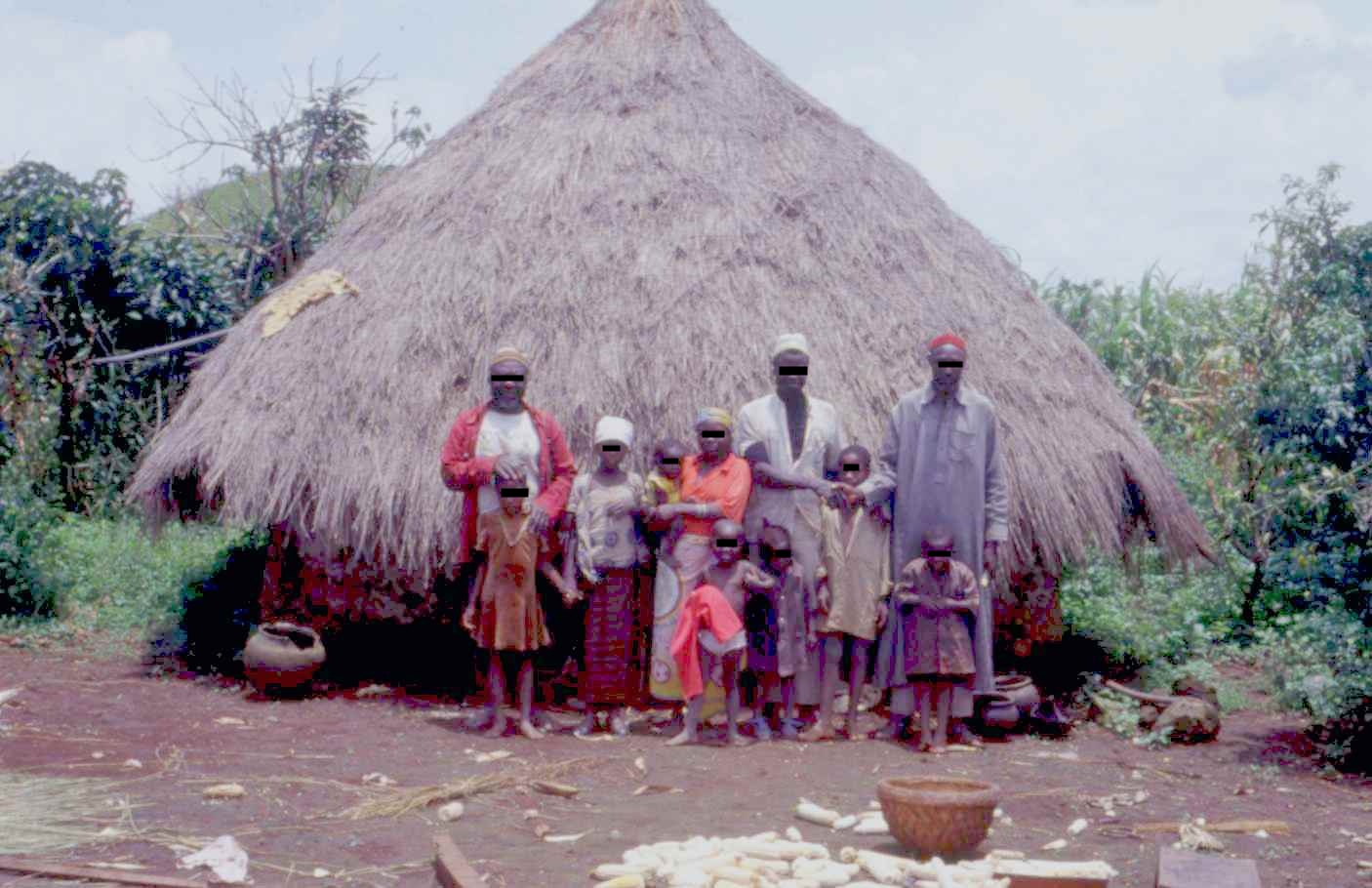

Supplement: Additional file 8 — "Test-person" OS with his family in front of their home. Pictures from Bolo and Galim: the village, villagers and the breeding sites are shown. [file 1756-3305-3-53-S8.JPEG]
